# Association Between the Physician Quality Score in the Merit-Based Incentive Payment System and Hospital Performance in Hospital Compare in the First Year of the Program

**DOI:** 10.1001/jamanetworkopen.2021.18449

**Published:** 2021-08-03

**Authors:** Laurent G. Glance, Caroline P. Thirukumaran, Changyong Feng, Stewart J. Lustik, Andrew W. Dick

**Affiliations:** 1 Department of Anesthesiology and Perioperative Medicine, University of Rochester School of Medicine, Rochester, New York; 2 Department of Public Health Sciences, University of Rochester School of Medicine, Rochester, New York; 3 RAND Health, RAND, Boston, Massachusetts; 4 Department of Orthopedics, University of Rochester School of Medicine, Rochester, New York; 5 Department of Biostatistics and Computational Biology, University of Rochester School of Medicine, Rochester, New York

## Abstract

**Importance:**

The scientific validity of the Merit-Based Incentive Payment System (MIPS) quality score as a measure of hospital-level patient outcomes is unknown.

**Objective:**

To examine whether better physician performance on the MIPS quality score is associated with better hospital outcomes.

**Design, Setting, and Participants:**

This cross-sectional study of 38 830 physicians used data from the Centers for Medicare & Medicaid Services (CMS) Physician Compare (2017) merged with CMS Hospital Compare data. Data analysis was conducted from September to November 2020.

**Main Outcomes and Measures:**

Linear regression was used to examine the association between physician MIPS quality scores aggregated at the hospital level and hospitalwide measures of (1) postoperative complications, (2) failure to rescue, (3) individual postoperative complications, and (4) readmissions.

**Results:**

The study cohort of 38 830 clinicians (5198 [14.6%] women; 12 103 [31.6%] with 11-20 years in practice) included 6580 (17.2%) general surgeons, 8978 (23.4%) orthopedic surgeons, 1617 (4.2%) vascular surgeons, 582 (1.5%) cardiac surgeons, 904 (2.4%) thoracic surgeons, 18 149 (47.4%) anesthesiologists, and 1520 (4.0%) intensivists at 3055 hospitals. The MIPS quality score was not associated with the hospital composite rate of postoperative complications. MIPS quality scores for vascular surgeons in the 11th to 25th percentile, compared with those in the 51st to 100th percentile, were associated with a 0.55–percentage point higher hospital rate of failure to rescue (95% CI, 0.06-1.04 percentage points; *P* = .03). MIPS quality scores for cardiac surgeons in the 1st to 10th percentile, compared with those in the 51st to 100th percentile, were associated with a 0.41–percentage point higher hospital coronary artery bypass graft (CABG) mortality rate (95% CI, 0.10-0.71 percentage points; *P* = .01). MIPS quality scores for cardiac surgeons in the 1st to 10th percentile and 11th to 25th percentile, compared with those in the 51st to 100th percentile, were associated with 0.65–percentage point (95% CI, 0.013-1.16 percentage points; *P* = .02) and 0.48–percentage point (95% CI, 0.07-0.90 percentage points; *P* = .02) higher hospital CABG readmission rates, respectively.

**Conclusions and Relevance:**

In this study, better performance on the physician MIPS quality score was associated with better hospital surgical outcomes for some physician specialties during the first year of MIPS.

## Key Points

**Question:**

Are higher scores on the Merit-Based Incentive Payment System (MIPS) for physicians associated with hospital-level patient outcomes?

**Findings:**

In this cross-sectional study of 38 830 clinicians, physician MIPS quality scores were not associated with the hospital’s overall rate of postoperative complications and were not generally associated with the hospital’s failure-to-rescue rate. However, low MIPS quality scores for cardiac surgeons were associated with higher hospital-level rates of coronary artery bypass graft mortality and readmissions.

**Meaning:**

The results of this exploratory study provide only limited evidence to support the validity of MIPS for measuring physician performance.

## Teaser

This cross-sectional study examines whether better physician performance on the Merit-Based Incentive Payment System (MIPS) is associated with better hospital outcomes.

## Introduction

Performance measurement is the centerpiece of the Center for Medicare & Medicaid Services (CMS) efforts to redesign the US health care system to deliver better patient outcomes at a lower cost. Under the 2015 Medicare Access and Children’s Reauthorization Act, CMS created the Quality Payment Program, which mandates that eligible clinicians participate in either the Merit-Based Incentive Payment System (MIPS) or Advanced Alternative Payment Models. Physicians, as either individuals or groups of physicians, are evaluated in the MIPS using a composite score between 0 and 100 points based on quality, improvement activities, and promoting interoperability. They can receive a maximum of 60 points for quality (10 points for each of 6 measures).^1^

The validity of the quality component of the MIPS score for comparing clinician performance has been challenged for several reasons.^2^ First, although physicians are required to report on 6 quality measures, they may select any 6 measures from the list of 271 available MIPS measures.^1^ Unlike Hospital Compare, in which hospital performance is rated using a standard set of uniform metrics, such as mortality and readmissions, physician performance in MIPS is measured using a composite score based on self-selected metrics that vary between physicians. Second, physicians are free to report the measures on which they perform best, rather than those that may best reflect their overall quality of care.^2^ Third, of these 6 measures, only 1 is required to be an outcome measure, while the others can be process measures.^1^ Process measures only reflect quality of care if they are anchored in best practices that lead to better outcomes. However, most recommendations in clinical practice guidelines are based only on expert opinion rather than experimental evidence.^3,4^ Fourth, physicians can choose to report either as individuals or as groups, and specialty physicians reporting as part of a multispecialty group may report measures that do not apply to their specialty.^2^

CMS has spent $1.3 billion on quality measure development over the last 10 years.^5^ In 2014, physician practices in the US spent 15 hours per week reporting quality measures, at an annual cost of $15.4 billion.^6^ Fewer than one-third of physician practices believe that physician performance measures are at least moderately associated with quality of care.^6^ Two-thirds of the MIPS measures used to evaluate ambulatory care were rated as not valid or of uncertain validity using criteria developed by the American College of Physicians.^7^ To our knowledge, the empirical validity of the MIPS quality score has not been previously evaluated for surgical care. This study aims to examine the empirical validity of the MIPS quality component by examining the association between the physician MIPS quality score and hospital-level postoperative outcomes for general surgeons, surgical subspecialties, anesthesiologists, and intensivists. This information may prove useful for informing CMS’s effort to introduce new value pathways in MIPS that focus on improving population health.^8^

## Methods

### Data Sources

This study was conducted using data from the publicly available CMS Physician Compare data sets (2017),^9^ CMS Hospital Compare data sets (2016-2018),^10^ CMS Physician and Other Supplier data set (2017),^11^ and the CMS Impact Files data set (2017).^12^ These data sets include information on physician demographic characteristics (gender, graduation year, primary specialty),^9^ aggregate measures of physician case mix (number of Medicare beneficiaries, age distribution, comorbidities, mean Hierarchical Condition Category [HCC] score, dual eligibility, and race and ethnicity),^11^ hospital characteristics (hospital size, resident-to-bed ratio, geographic region),^12^ physician performance on MIPS quality indicators (quality scores, reporting source [individual, group, other]),^9^ and hospital performance (Patient Safety and Adverse Events Composite; deaths among surgical inpatients with serious treatable complications, ie, the failure-to-rescue rate; coronary artery bypass grafting [CABG] mortality; complications after hip and knee replacements; and individual postoperative complications, ie, respiratory failure, sepsis, acute kidney injury, postoperative hemorrhage).^10^ National Provider Identifier numbers and hospital CMS Certification Number identifiers were used to link data sets. The 2017 physician performance data are the most recent available data with physician identifiers as of November 2020 and were the basis for physician payment adjustments in 2019.^13^ For this analysis, the first hospital listed in the Physician Compare data set was used to link the physician data set with the CMS Hospital Compare data set when more than 1 hospital was listed for a physician.

The institutional review board of the University of Rochester School of Medicine and Dentistry reviewed this study protocol and determined that this research meets federal and university criteria for exempt research. The findings of this study are reported following the Strengthening the Reporting of Observational Studies in Epidemiology (STROBE) reporting guideline.^22^

### Study Sample

We identified 93 269 physicians with the following primary specialties: general surgery, vascular surgery, orthopedics, cardiac surgery, thoracic surgery, anesthesiology, and critical care medicine (eFigure 1 in the Supplement). We limited our analysis to physicians who had more than 5 years in practice to exclude resident physicians (n = 8438). Physicians with missing hospital affiliation (n = 588) were also excluded. For each specialty group (eg, general surgeons) within a hospital, we calculated the proportion of physicians with missing quality scores or missing case volumes. We treated physician quality scores equal to 0 as missing because in the first year of MIPS, physicians who submitted any quality measure information received a minimum quality score of 3.^14^ Hospitals with 20% or more missing data were excluded from the analysis. We did not use multiple imputation because it was not likely that the missing data on quality scores would only depend on the observed data and meet the missing at random assumption.^15,16^ We also excluded hospitals that did not report measures in Hospital Compare. The analytic data set consisted of 38 830 physicians affiliated with 3055 hospitals.

### Statistical Analysis

The primary outcome was the hospital composite rate of serious postoperative complications (represented by the Agency for Healthcare Research and Quality [AHRQ] Patient Safety and Adverse Events Composite).^17^ We used linear regression to examine the association between the hospital composite complication rate and the hospital-level physician MIPS quality score. If physicians had both a physician and group MIPS quality score available, we used the physician-level score. We first specified hospital-level physician MIPS scores within each hospital by taking a weighted average of the MIPS scores for physicians affiliated with a hospital based on each physician’s share of the hospital case volume (each physician’s quality score was multiplied by the ratio of their case volume divided by the total surgical case volume). We used a physician’s total number of unique Medicare beneficiaries as a proxy for each physician’s surgical caseload because we did not have access to actual surgical case volumes. We then specified the hospital-level physician MIPS score as a categorical variable to account for the nonlinear association between the hospital composite rate of serious postoperative complications and the hospital-level physician MIPS score: 1st to 10th percentile, 11th to 25th percentile, 26th to 50th percentile, and 51st to 100th percentile (reference category). We performed separate analyses for each of the physician specialties (general surgery, vascular surgery, orthopedics, cardiac surgery, thoracic surgery, anesthesiology, and critical care medicine). We repeated this main analysis to examine the association between the failure-to-rescue rate (deaths among surgical inpatients with serious treatable complications) and the hospital-level physician MIPS quality score.^17^ We also estimated standardized effect sizes by normalizing the dependent variable so that, eg, the difference in the hospital complication rate between general surgeon MIPS scores in the 1st to 10th percentile and the 51st to 100th percentile (reference category) was 1 SD of the outcome (eg, complication rates for general surgeons) when the standardized coefficient was 1. Standardized coefficients greater than 0.2 were considered clinically meaningful effect sizes.^18^

We then performed several secondary analyses using several secondary end points. First, we examined the association between the hospital-level physician MIPS quality scores for cardiac surgeons, anesthesiologists, and intensivists and (1) hospital CABG mortality and (2) readmission rates.^17^ Second, we examined the association between the hospital-level MIPS quality score for orthopedic surgeons, anesthesiologists, and intensivists with hospital rates of complications after hip and knee replacements.^17^ Finally, we examined the association between the hospital-level physician MIPS quality score and some of the complications included in the AHRQ Patient Safety and Adverse Events Composite: postoperative respiratory failure, postoperative sepsis, postoperative acute kidney injury, and postoperative hemorrhage for each of the physician groups.

Data management and statistical analyses were performed using Stata SE/MP version 16.1 (StataCorp). All statistical tests were 2-tailed, and *P* < .05 was considered significant. Because of the large number of analyses, using a significance threshold of .05 could lead to a high risk of falsely concluding that there is a significant association between MIPS scores and 1 or more hospital outcomes tested.^19^ We decided a priori not to correct for multiple comparisons as a conservative strategy^20^ to avoid falsely concluding that there was no association between MIPS scores and hospital outcomes. We believe that such a conservative approach is justified given that the MIPS quality scores may be associated with some domains of hospital outcomes (eg, CABG mortality) and not others (postoperative sepsis) or for some physician specialties (eg, cardiac surgery) and not others (eg, orthopedic surgery). In addition, this approach is reasonable because the effect sizes are expected to be small considering that we are examining the association between global measures of hospital outcomes that are influenced by several surgical specialties (eg, failure-to-rescue rates) and MIPS scores based on a single surgical specialty (eg, general surgeons). Because there is no accepted threshold for judging the strength of the association between a new measure (the MIPS quality score) and accepted measures (ie, CABG mortality rate),^21^ we decided to use the predefined value of 0.2 as the minimum threshold for a small standardized effect size.^18^

## Results

### Physician, Hospital, and Patient Characteristics

The study was based on 38 330 physicians (5198 [14.6%] women; 12 103 [31.6%] with 11-20 years in practice) affiliated with 3055 hospitals (Table). Of the 38 330 physicians in the sample, 6850 (17.2%) were general surgeons, 8978 (23.4%) were orthopedic surgeons, 1617 (4.2%) were vascular surgeons, 18 149 (47.4%) were anesthesiologists, and 1520 (4.0%) were intensivists. Overall, 19 940 physicians (51.3%) were in practice for 21 years or more (based on the year of medical school graduation). More than half of physicians (22 625 [58.3%]) cared for more than 200 Medicare beneficiaries. The mean (SD) age of the Medicare beneficiaries in physician practices was 71.1 (3.2) years, and the mean percentage of patients in physician practices with ischemic heart disease, congestive heart failure, and chronic kidney disease was 42.7% (13.8), 27.1% (14.0), and 41.4% (14.0), respectively.

**Table.** Physician, Physician Practice, and Hospital Characteristics

Characteristic	No. (%)
Physician characteristics	
No.	38 330
Specialty	
General surgery	6580 (17.2)
Orthopedic surgery	8978 (23.4)
Vascular surgery	1617 (4.2)
Cardiac surgery	582 (1.5)
Thoracic surgery	904 (2.4)
Anesthesiology	18 149 (47.4)
Critical care medicine	1520 (4.0)
Years in practice	
6-10	6287 (16.4)
11-20	12 103 (31.6)
21-30	11 093 (28.9)
≥31	8847 (23.1)
Women	5198 (14.6)
Men	33 132 (86.4)
Physician practice characteristics	
No. of Medicare beneficiaries	
11-200	15 705 (41)
201-300	8273 (21.6)
301-400	5158 (13.5)
≥401	9194 (24)
Age of Medicare beneficiaries, mean (SD), y	71.1 (3.2)
Patient risk score, mean (SD)	1.8 (0.9)
Comorbidities, mean (SD), %^a^	
Ischemic heart disease	42.7 (13.8)
Congestive heart failure	27.1 (14.0)
Stroke	8.5 (5.5)
Atrial fibrillation	16.8 (8.2)
COPD	22.2 (10.0)
Chronic kidney disease	41.4 (14.0)
Diabetes	36.8 (10.0)
Dual eligible patients, mean (SD), %^a^	23.8 (13.1)
Race/ethnicity, mean (SD), %^a^	
Black	14.4 (13.8)
Hispanic	11.2 (12.3)
Score source	
Individual	5218 (13.6)
Group	27 598 (72)
Alternative Payment Model	3873 (10.1)
Other	1641 (4.3)
Hospital characteristics	
No.	3055
Size	
Small (≤99 beds)	796 (26.1)
Medium (100-399 beds)	1381 (45.2)
Large (≥400 beds)	878 (28.7)
Resident-to-bed ratio, mean (SD)	0.07 (0.17)
Region	
New England	108 (3.5)
Middle Atlantic	287 (9.4)
South Atlantic	480 (15.7)
East North Central	413 (13.5)
East South Central	208 (6.8)
West North Central	218 (7.1)
West South Central	360 (11.8)
Mountain	173 (5.7)
Pacific	269 (8.8)
Puerto Rico	2 (0.1)
Missing	537 (17.6)

Abbreviation: COPD, chronic obstructive pulmonary disease.

^a^ These represent the mean of the percentage of patients with a specific characteristic (eg, ischemic heart disease) cared for by individual physicians.

Most hospitals were medium in size (100-399 beds) or larger. The distribution of physician MIPS quality scores was skewed, with 50% of the quality scores equal to or greater than 92 (eFigure 2 in the Supplement).

### MIPS Quality Score and Postoperative Complication Composite

**Figure 1.**
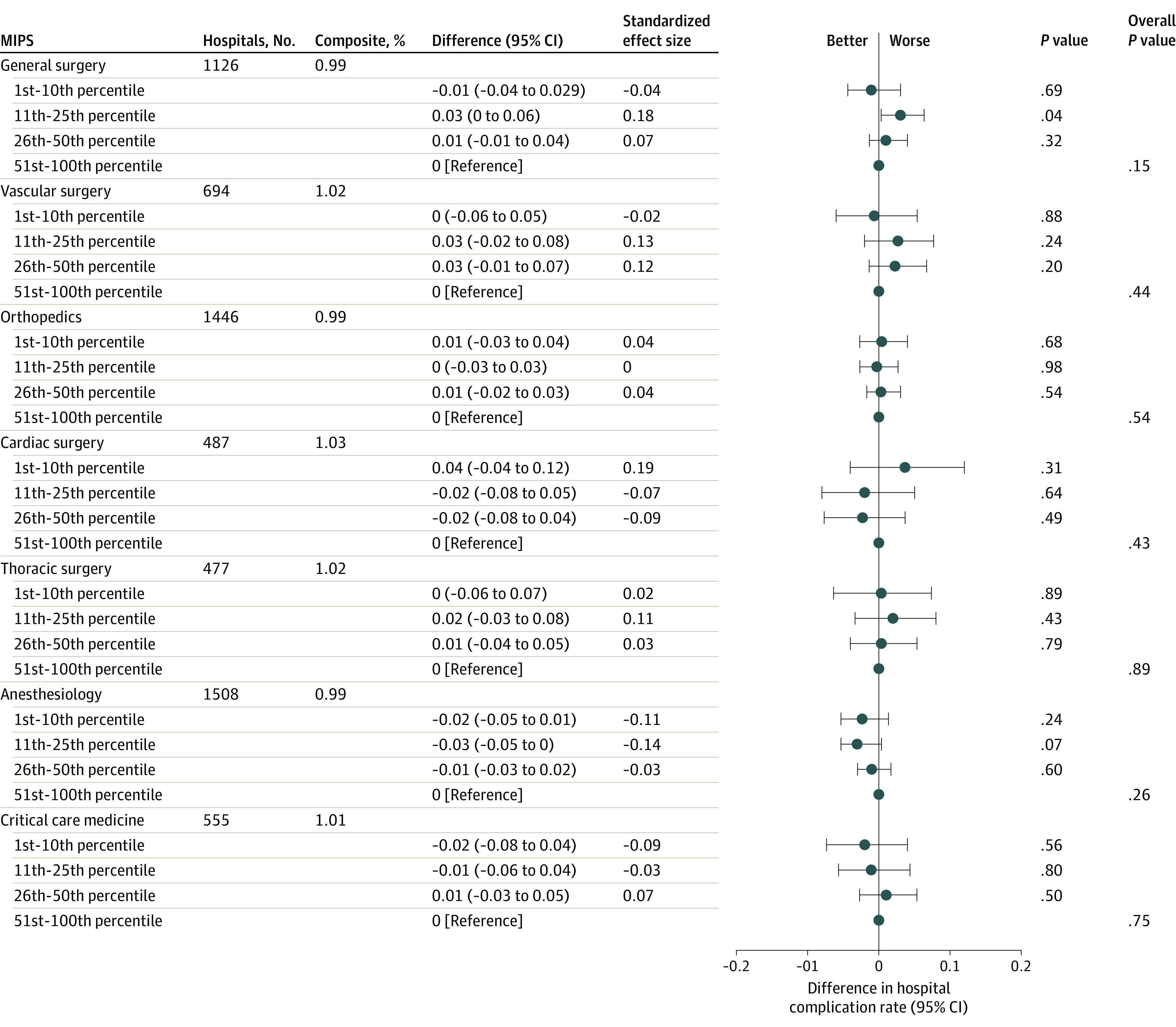
Association Between Physician Quality Merit-Based Incentive Payment System (MIPS) Scores and Patient Safety and Adverse Events Composite Difference refers to the percentage point difference between the MIPS group (eg, 1st-10th percentile) and the reference category (51st-100th percentile). Standardized effect size refers to the standardized coefficient for each quartile, such that a standardized coefficient of 1 for the 1st to 10th percentile indicates the percentage point difference between the 1st to 10th percentile and the 51st to 100th percentile is 1 SD (based on the overall distribution of the hospital complication rate).

The hospital-level weighted mean of physician MIPS quality scores (hereafter referred to as the MIPS quality score) was not associated with the hospital rate of postoperative complications (AHRQ Patient Safety and Adverse Events Composite) (eg, general surgeons in 1st-10th percentile vs those in 51st-100th: difference, −0.01; 95% CI, −0.04 to 0.03; standardized effect size, −0.04; *P* = .69) (Figure 1; eFigures 3-6 in the Supplement).

### MIPS Quality Score and Failure-to-Rescue

**Figure 2.**
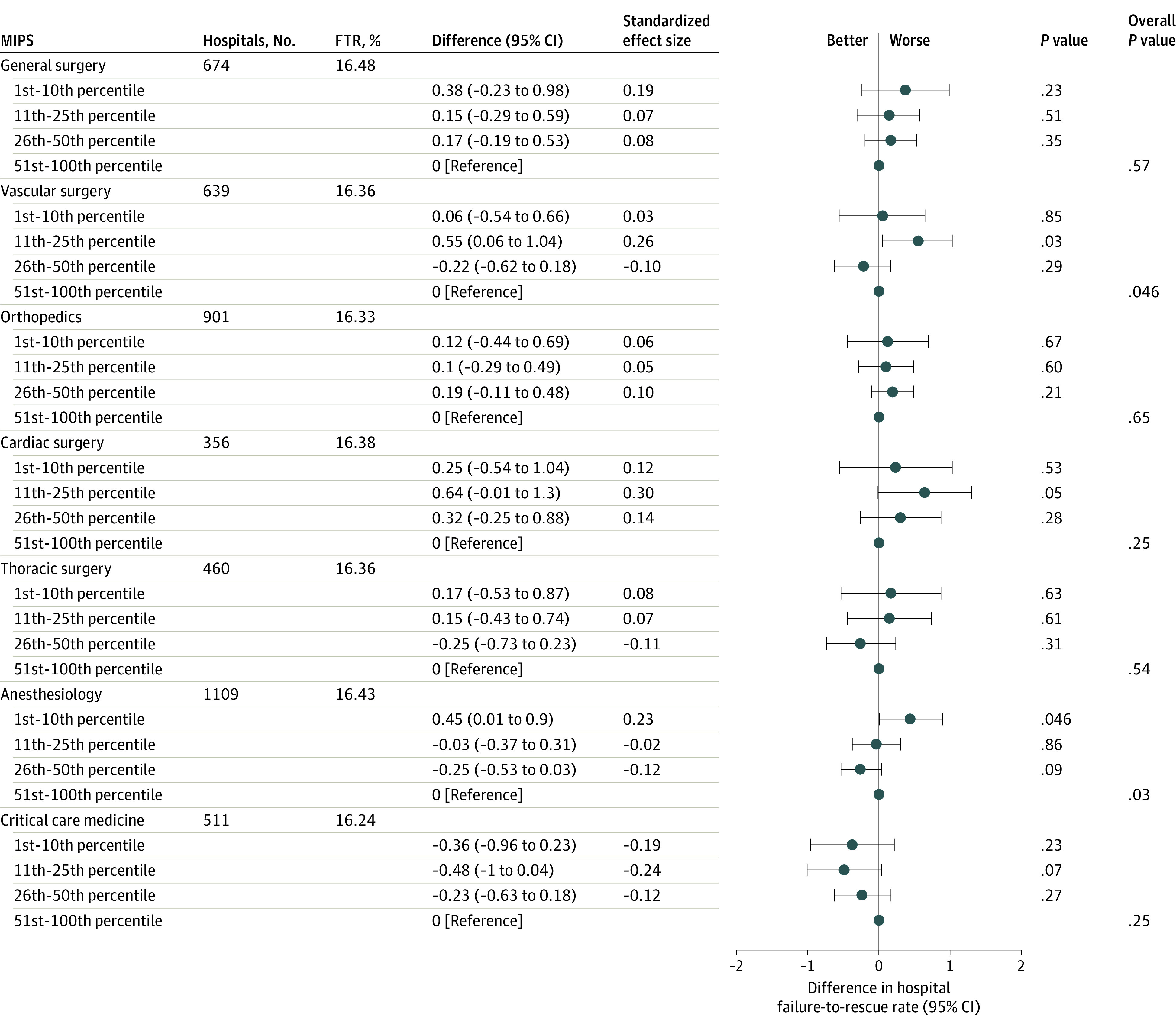
Association Between Physician Quality Merit-Based Incentive Payment System (MIPS) Scores and Failure-to-Rescue (FTR) Rate Difference refers to the percentage point difference between the MIPS group (eg, 1st-10th percentile) and the reference category (51st-100th percentile). Standardized effect size refers to the standardized coefficient for each quartile, such that a standardized coefficient of 1 for the 1st to 10th percentile indicates the percentage point difference between the 1st to 10th percentile and the 51st to 100th percentile is 1 SD (based on the overall distribution of the hospital complication rate).

MIPS quality scores for vascular surgeons in the 11th to 25th percentile were associated with a 0.55–percentage point higher failure-to-rescue rate (95% CI, 0.06-1.04 percentage points; *P* = .03) compared with MIPS quality scores for vascular surgeons in the 51st to 100th percentile (Figure 2). MIPS quality scores for anesthesiologists in the 1st to 10th percentile were associated with a 0.45–percentage point higher rate of complications (95% CI, 0.01-0.90 percentage points; *P* = .046) compared with MIPS quality scores for anesthesiologists in the 51st to 100th percentile.

### MIPS Quality Score and Specialty-Specific Outcomes

**Figure 3.**
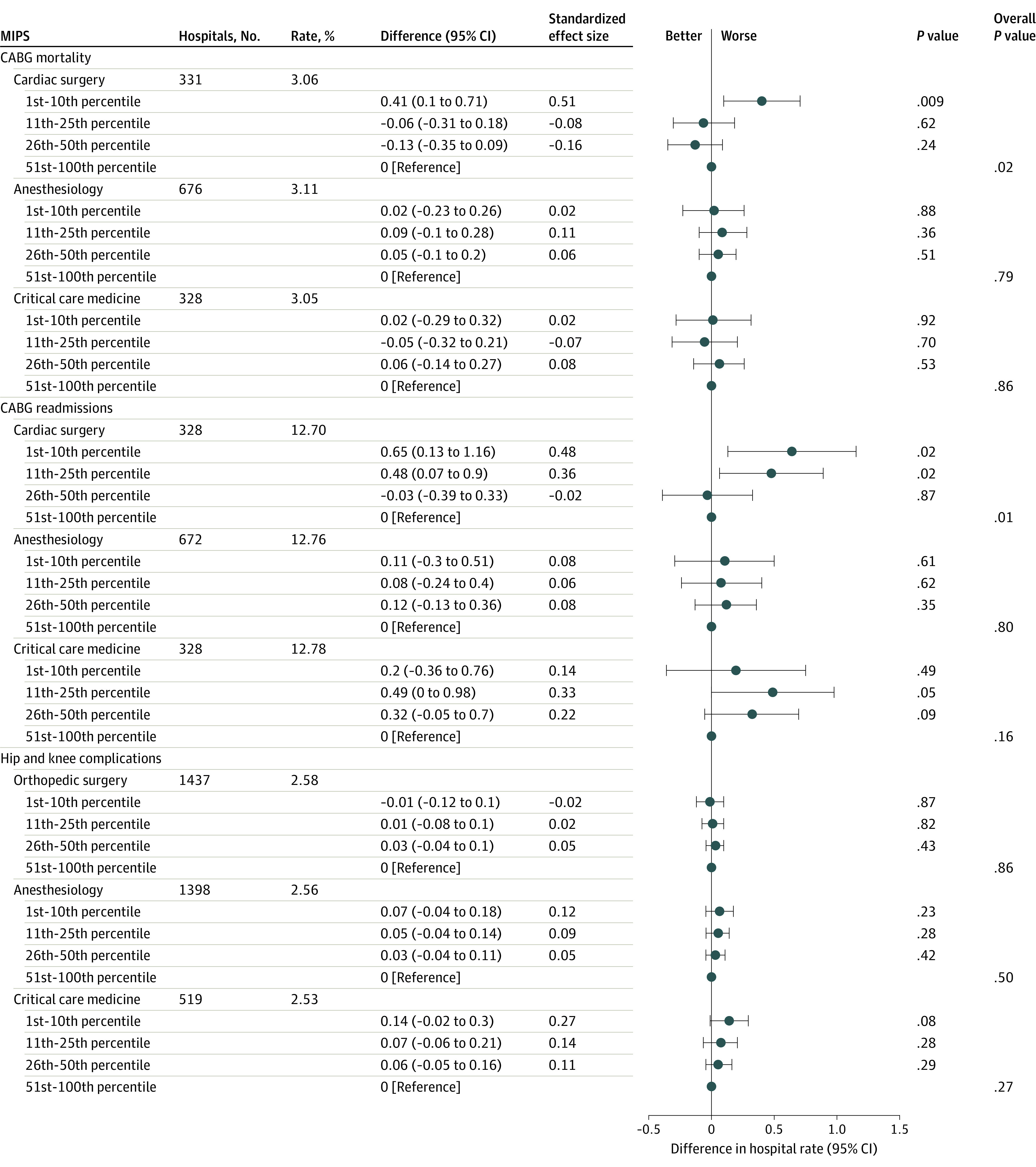
Association Between Physician Quality Merit-Based Incentive Payment System (MIPS) Scores and Hospital Outcomes for Coronary Artery Bypass Graft (CABG) and Hip or Knee Replacement Difference refers to the percentage point difference between the MIPS group (eg, 1st-10th percentile) and the reference category (51st-100th percentile). Standardized effect size refers to the standardized coefficient for each quartile, such that a standardized coefficient of 1 for the 1st to 10th percentile indicates the percentage point difference between the 1st to 10th percentile and the 51st to 100th percentile is 1 SD (based on the overall distribution of the hospital complication rate).

The MIPS quality score for cardiac surgeons was associated with CABG mortality and CABG readmissions (Figure 3). MIPS quality scores for cardiac surgeons in the 1st to 10th percentile were associated with a 0.41–percentage point higher CABG mortality rate (95% CI, 0.10-0.71 percentage points; *P* = .01) compared with MIPS quality scores for cardiac surgeons in the 51st to 100th percentile. MIPS quality scores for cardiac surgeons in the 1st to 10th percentile and 11th to 25th percentile were associated with a 0.65–percentage point (95% CI, 0.01-1.16 percentage points; *P* = .02) and a 0.48–percentage point (95% CI, 0.07-0.90 percentage points; *P* = .02) higher CABG readmission rates compared with MIPS quality scores for cardiac surgeons in the 51st to 100th percentile, respectively. MIPS quality scores for anesthesiologists and intensivists were not associated with hospital CABG mortality or readmission rates (Figure 3).

For hip and knee complications, MIPS quality scores for orthopedic surgeons were not associated with hospital rates of hip and knee complications (Figure 3). MIPS quality scores for anesthesiologists and intensivists were also not associated with hip and knee complications.

## Discussion

We found limited evidence to support the empirical validity of the MIPS quality component for surgical patients. MIPS quality scores for vascular surgeons and anesthesiologists were associated with small but clinically meaningful differences in 1 global measure of hospital performance, the failure-to-rescue rate. MIPS quality scores for other surgical specialties and intensivists were not associated with either failure-to-rescue rates or postoperative complications. When we focused instead on specific surgeries, we found that MIPS quality scores for cardiac surgeons were associated with small and clinically meaningful differences in hospital rates of CABG 30-day mortality and readmissions, while orthopedic surgeon MIPS scores were not associated with hospital rates of complications after hip and knee replacements. Finally, when we examined specific complications included in the postoperative complication measure, we found that lower MIPS quality scores for general surgeons and orthopedic surgeons were associated with higher rates of postoperative respiratory failure, while lower MIPS quality scores were associated with higher rates of postoperative sepsis for thoracic surgeons.

It is perhaps not surprising that physician MIPS scores are, at best, only weakly associated with hospital performance. There are several possible explanations for this, including the unusually high number of physicians with very high MIPS scores, the preponderance of process measures as opposed to outcome measures, the lack of specialty-specific mandatory measurement sets, the absence of a fixed data submission period, and scoring adjustments by CMS unrelated to physician performance.

First, the concentration of scores at the top end of the distribution is consistent with a recent report that 40% of MIPS measures are topped out, meaning that most clinicians score near the top of the distribution of these performance scores.^23^ By contrast, most measures of hospital performance are normally distributed.^24^ The high proportion of physicians achieving near-perfect quality scores may be due to several factors. Because physicians select which measures to report, it is likely that they will choose only those measures on which they perform best. In addition, physicians are only required to report 1 outcome measure. The remainder can be process measures that, unlike patient outcome measures, are more directly under physicians’ control and can be manipulated to achieve higher scores.^25^ At the other end of the score distribution, physicians submitting data on a measure for which CMS cannot establish a scoring curve received only 3 of a possible 10 points even if their performance on the measure was excellent.^2^ Thus, very low MIPS scores may not necessarily reflect below-average physician performance, while the high end of the scoring distribution may be too narrowly concentrated to reflect meaningful differences in performance.

Second, most MIPS measures are process measures, and better performance on process measures will only lead to better patient outcomes if these measures reflect best clinical practices. Ideally, process measures should be based on recommendations in clinical practice guidelines supported by strong scientific evidence. But, even if most process measures were based on clinical practice guidelines, most of the recommendations in clinical practice guidelines are based on expert opinion and not on high-quality evidence.^4,26,27,28,29^

Third, there is no mandatory specialty-specific set of performance measures on which each physician must report. Physicians may submit any measures approved for MIPS reporting, including measures outside of their specialty-specific measure set.^2^ For example, a smoking cessation measure is unlikely to reflect a surgeon’s or anesthesiologist’s technical proficiency and risk of complications. Fourth, given that physicians can decide to submit data for 90 days or as long as a full year, performance can, in theory, be optimized by choosing the best time period to report. Although prohibited by CMS, it is also theoretically possible for physicians to cherry-pick patients because they are only required to submit data on 50% of eligible patients for a specific measure.^1,30^ Finally, 2 physicians may report identical scores on the same performance measure but will receive a different number of points if they use different data sources (eg, claims data vs electronic health record data) when the score distributions differ across data types.^2^

To our knowledge, ours is the first study to report on the association between the MIPS quality score and patient outcomes. Few studies have been published on MIPS, and these have focused on the association of caring for patients with social disadvantage with MIPS scores and reimbursements.^31,32,33^ One study did examine the association between patient-reported experiences in the Physician Quality Reporting System (which is the precursor of the quality component of MIPS)^34^ and surgical outcomes in the American College of Surgeons National Surgical Quality Improvement Program. This study reported that better patient-related experiences across 2 domains were associated with lower rates of complications, readmissions, and reoperations.^35^

### Limitations

Our study has several limitations. Most importantly, this exploratory analysis did not control for multiple comparisons to avoid falsely concluding that there is no association between the MIPS quality score and patient outcomes. Although it is appropriate not to correct for multiple comparisons in an exploratory analysis, not doing so does increase the risk of false-positive inferences.^19^ Second, although our approach is consistent with that used by the National Quality Forum to empirically validate new measures by examining the association of a new measure with existing measures,^21^ our analysis may be biased toward the null because it was not possible to limit our analyses to physician-specific cohorts (ie, outcomes for vascular surgery patients in the case of vascular surgeons). Third, we constructed a hospital-level composite for the physician-level MIPS score by weighting the MIPS scores by each physician’s number of unique Medicare beneficiaries instead of their surgical case volume, which was not available in our data. The physician weights may less accurately reflect surgical case volumes for anesthesiologists compared with surgeons because anesthesiologists deliver anesthesia for cases outside the operating room. Fourth, excluding hospitals in which more than 20% of the physicians did not submit quality measures may limit the generalizability of our findings. Fifth, because we examined the association between physician MIPS quality scores and hospital outcomes based on the first hospital listed in the Physician Compare data set, the MIPS scores for physicians who worked at more than 1 hospital were not attributed to all of the hospitals where they worked, which could have biased our results toward the null. Sixth, we did not include certified nurse anesthetists (CRNAs) in our analysis. Because CRNAs deliver anesthesia without the supervision of a physician anesthesiologist in as much as 21% of cases,^36^ excluding CRNAs from our analysis may have also biased the results of our analysis examining the association between anesthesiologist MIPS scores and hospital outcomes. Seventh, a stronger association between the MIPS quality score and surgical outcomes cannot be ruled out without first examining the association of the MIPS quality score with patient outcomes using patient-level instead of hospital-level data.

## Conclusions

In this cross-sectional study, we found limited evidence to show that better performance on the physician MIPS quality score was associated with lower rates of hospital complications in surgical patients during the first year of MIPS. Concerns have been raised that MIPS may not sufficiently incentivize physicians to deliver high-value care.^13^ However, the main problem with MIPS may not be whether the incentives are large enough to influence physician behavior but rather whether the MIPS quality score is scientifically valid and measures physicians’ contribution to outcomes.

## Notes

Supplement. **eAppendix.** Results of Secondary Analyses **eFigure 1. **Flowchart **eFigure 2. **Distribution of Physician MIPS Quality Scores **eFigure 3. **Association Between Physician Quality MIPS Scores and Postoperative Respiratory Failure **eFigure 4. **Association Between Physician Quality MIPS Scores and Postoperative Sepsis **eFigure 5. **Association Between Physician Quality MIPS Scores and Postoperative Acute Kidney Injury **eFigure 6. **Association Between Physician Quality MIPS Scores and Postoperative Bleeding

## References

[1] Centers for Medicare & Medicaid Services. 2017 MIPS quality performance category fact sheet. October 31, 2018. Accessed June 29, 2021. https://www.hhs.gov/guidance/document/2017-mips-quality-performance-category-fact-sheet-0

[2] Rathi VK, McWilliams JM. First-year report cards from the Merit-Based Incentive Payment System (MIPS): what will be learned and what next? JAMA. 2019;321(12):1157-1158. doi:10.1001/jama.2019.1295 30830150

[3] Tricoci P, Allen JM, Kramer JM, Califf RM, Smith SC Jr. Scientific evidence underlying the ACC/AHA clinical practice guidelines. JAMA. 2009;301(8):831-841. doi:10.1001/jama.2009.205 19244190

[4] Fanaroff AC, Califf RM, Windecker S, Smith SC Jr, Lopes RD. Levels of evidence supporting American College of Cardiology/American Heart Association and European Society of Cardiology Guidelines, 2008-2018. JAMA. 2019;321(11):1069-1080. doi:10.1001/jama.2019.1122 30874755 PMC6439920

[5] Wadhera RK, Figueroa JF, Joynt Maddox KE, Rosenbaum LS, Kazi DS, Yeh RW. Quality measure development and associated spending by the Centers for Medicare & Medicaid Services. JAMA. 2020;323(16):1614-1616. doi:10.1001/jama.2020.1816 32343321 PMC7189223

[6] Casalino LP, Gans D, Weber R, . US physician practices spend more than $15.4 billion annually to report quality measures. Health Aff (Millwood). 2016;35(3):401-406. doi:10.1377/hlthaff.2015.1258 26953292

[7] MacLean CH, Kerr EA, Qaseem A. Time out—charting a path for improving performance measurement. N Engl J Med. 2018;378(19):1757-1761. doi:10.1056/NEJMp1802595 29668361

[8] Quality Payment Program. 2020 Quality Payment Program proposed rule overview factsheet with request for information for 2021. Accessed October 2, 2020. https://qpp-cm-prod-content.s3.amazonaws.com/uploads/594/2020%20QPP%20Proposed%20Rule%20Fact%20Sheet.pdf

[9] Centers for Medicare & Medicaid Services. Physician Compare datasets. Accessed October 10, 2021. https://data.medicare.gov/data/physician-compare

[10] Centers for Medicare & Medicaid Services. Hospital Compare datasets. Accessed October 10, 2021. https://data.medicare.gov/data/hospital-compare

[11] Centers for Medicare & Medicaid Services. Medicare and other supplier National Provider Identifier aggregate report. Accessed October 10, 2021. https://data.cms.gov/Medicare-Physician-Supplier/Medicare-Physician-and-Other-Supplier-National-Pro/n5qc-ua94

[12] National Bureau of Economic Research. CMS Impact File Hospital Inpatient Prospective Payment System (IPPS). Accessed October 10, 2021. https://data.nber.org/data/cms-impact-file-hospital-inpatient-prospective-payment-system-ipps.html

[13] Apathy NC, Everson J. High rates of partial participation in the first year of the Merit-Based Incentive Payment System. Health Aff (Millwood). 2020;39(9):1513-1521. doi:10.1377/hlthaff.2019.01648 32897783 PMC7720898

[14] Quality Payment Program. Explore measures and activities. Accessed October 4, 2020. https://qpp.cms.gov/mips/explore-measures?tab=qualityMeasures&py=2017

[15] Donders AR, van der Heijden GJ, Stijnen T, Moons KG. Review: a gentle introduction to imputation of missing values. J Clin Epidemiol. 2006;59(10):1087-1091. doi:10.1016/j.jclinepi.2006.01.014 16980149

[16] Glance LG, Osler TM, Mukamel DB, Meredith W, Dick AW. Impact of statistical approaches for handling missing data on trauma center quality. Ann Surg. 2009;249(1):143-148. doi:10.1097/SLA.0b013e31818e544b 19106690

[17] Centers for Medicare & Medicaid Services. Outcome measures. Accessed October 29, 2020. https://www.cms.gov/Medicare/Quality-Initiatives-Patient-Assessment-Instruments/HospitalQualityInits/OutcomeMeasures

[18] Cohen J. Statistical Power Analysis for the Behavioral Sciences. Lawrence Erlbaum Associates; 1988.

[19] Cao J, Zhang S. Multiple comparison procedures. JAMA. 2014;312(5):543-544. doi:10.1001/jama.2014.9440 25096694

[20] Ingraham AM, Cohen ME, Bilimoria KY, . Association of surgical care improvement project infection-related process measure compliance with risk-adjusted outcomes: implications for quality measurement. J Am Coll Surg. 2010;211(6):705-714. doi:10.1016/j.jamcollsurg.2010.09.006 21109157

[21] Glance LG, Joynt Maddox K, Johnson K, . National Quality Forum guidelines for evaluating the scientific acceptability of risk-adjusted clinical outcome measures: a report from the National Quality Forum Scientific Methods Panel. Ann Surg. 2020;271(6):1048-1055. doi:10.1097/SLA.0000000000003592 31850998

[22] von Elm E, Altman DG, Egger M, Pocock SJ, Gøtzsche PC, Vandenbroucke JP; STROBE Initiative. Strengthening the Reporting of Observational Studies in Epidemiology (STROBE) statement: guidelines for reporting observational studies. BMJ. 2007;335(7624):806-808. doi:10.1136/bmj.39335.541782.AD 17947786 PMC2034723

[23] Golding LP, Nicola GN, Duszak R Jr, Rosenkrantz AB. The Quality measure crunch: how CMS topped out scoring and removal policies disproportionately disadvantage radiologists. J Am Coll Radiol. 2020;17(1 Pt B):110-117. doi:10.1016/j.jacr.2019.08.014 31918866

[24] Krumholz HM, Merrill AR, Schone EM, . Patterns of hospital performance in acute myocardial infarction and heart failure 30-day mortality and readmission. Circ Cardiovasc Qual Outcomes. 2009;2(5):407-413. doi:10.1161/CIRCOUTCOMES.109.883256 20031870

[25] Glance LG, Neuman M, Martinez EA, Pauker KY, Dutton RP. Performance measurement at a “tipping point”. Anesth Analg. 2011;112(4):958-966. doi:10.1213/ANE.0b013e31820e778d 21385976

[26] Duarte-García A, Zamore R, Wong JB. The evidence basis for the American College of Rheumatology practice guidelines. JAMA Intern Med. 2018;178(1):146-148. doi:10.1001/jamainternmed.2017.6680 29181496 PMC5833511

[27] Chauhan SP, Berghella V, Sanderson M, Magann EF, Morrison JC. American College of Obstetricians and Gynecologists practice bulletins: an overview. Am J Obstet Gynecol. 2006;194(6):1564-1572. doi:10.1016/j.ajog.2006.03.001 16731072

[28] Khan AR, Khan S, Zimmerman V, Baddour LM, Tleyjeh IM. Quality and strength of evidence of the Infectious Diseases Society of America clinical practice guidelines. Clin Infect Dis. 2010;51(10):1147-1156. doi:10.1086/656735 20946067

[29] Alseiari M, Meyer KB, Wong JB. Evidence underlying KDIGO (Kidney Disease: Improving Global Outcomes) guideline recommendations: a systematic review. Am J Kidney Dis. 2016;67(3):417-422. doi:10.1053/j.ajkd.2015.09.016 26526035

[30] Medicare program CY 2020 revisions to payment policies under the physician fee schedule and other changes to Part B payment policies. Accessed June 29, 2021. https://www.federalregister.gov/documents/2019/08/14/2019-16041/medicare-program-cy-2020-revisions-to-payment-policies-under-the-physician-fee-schedule-and-other

[31] Khullar D, Schpero WL, Bond AM, Qian Y, Casalino LP. Association between patient social risk and physician performance scores in the first year of the merit-based incentive payment system. JAMA. 2020;324(10):975-983. doi:10.1001/jama.2020.13129 32897345 PMC7489811

[32] Johnston KJ, Hockenberry JM, Wadhera RK, Joynt Maddox KE. Clinicians with high socially at-risk caseloads received reduced merit-based incentive payment system scores. Health Aff (Millwood). 2020;39(9):1504-1512. doi:10.1377/hlthaff.2020.00350 32897781

[33] Sandhu AT, Bhattacharya J, Lam J, . Adjustment for social risk factors does not meaningfully affect performance on Medicare’s MIPS clinician cost measures. Health Aff (Millwood). 2020;39(9):1495-1503. doi:10.1377/hlthaff.2020.00440 32897780

[34] Koltov MK, Damle NS. Health policy basics: physician quality reporting system. Ann Intern Med. 2014;161(5):365-367. doi:10.7326/M14-0786 24957469

[35] Liu JB, Pusic AL, Gibbons CJ, . Association of patient-reported experiences and surgical outcomes among group practices: retrospective cohort study. Ann Surg. 2020;271(3):475-483. doi:10.1097/SLA.0000000000003034 30188401

[36] Dulisse B, Cromwell J. No harm found when nurse anesthetists work without supervision by physicians. Health Aff (Millwood). 2010;29(8):1469-1475. doi:10.1377/hlthaff.2008.0966 20679649

