# Supplementary material for: Association Between the Physician Quality Score in the Merit-Based Incentive Payment System and Hospital Performance in Hospital Compare in the First Year of the Program
Source: JAMA Netw Open. 2021 Aug 3;4(8):e2118449. doi: 10.1001/jamanetworkopen.2021.18449 (PMC8335582; doi:10.1001/jamanetworkopen.2021.18449)
Supplement: Supplement. — eAppendix. Results of Secondary Analyses eFigure 1. Flowchart eFigure 2. Distribution of Physician MIPS Quality Scores eFigure 3. Association Between Physician Quality MIPS Scores and Postoperative Respiratory Failure eFigure 4. Association Between Physician Quality MIPS Scores and Postoperative Sepsis eFigure 5. Association Between Physician Quality MIPS Scores and Postoperative Acute Kidney Injury eFigure 6. Association Between Physician Quality MIPS Scores and Postoperative Bleeding [file jamanetwopen-e2118449-s001.pdf]

Supplemental Online Content

Glance LG, Thirukumaran CP, Feng C, Lustik SJ, Dick AW. Association between the physician quality score in the Merit-Based Incentive Payment System and hospital performance in Hospital Compare in the first year of the program. *JAMA Netw Open.* 2021;4(8):e2118449. doi:10.1001/jamanetworkopen.2021.18449

eAppendix. Results of Secondary Analyses

eFigure 1. Flowchart

eFigure 2. Distribution of Physician MIPS Quality Scores

eFigure 3. Association Between Physician Quality MIPS Scores and Postoperative Respiratory Failure

eFigure 4. Association Between Physician Quality MIPS Scores and Postoperative Sepsis

eFigure 5. Association Between Physician Quality MIPS Scores and Postoperative Acute Kidney Injury

eFigure 6. Association Between Physician Quality MIPS Scores and Postoperative Bleeding

This supplemental material has been provided by the authors to give readers additional information about their work.

© 2021 Glance LG et al. *JAMA Network Open.*

eAppendix. **Results of Secondary Analyses**

MIPS Quality Score and Components of Postoperative Complication Composite (respiratory failure, sepsis, acute kidney injury & postoperative hemorrhage)

The MIPS quality scores for general surgeons and orthopedic surgeons were associated with hospital rates of postoperative respiratory failure (Appendix Figure 3). In particular, MIPS quality scores for general surgeons in the 11th to 25th percentile were associated with a 0.76 percentage point higher rate of respiratory failure (95% CI: 0.26-1.25, P = 0.003) compared to MIPS quality scores for general surgeons in the 51st to 100th percentile. MIPS quality scores for orthopedic surgeons in the 1st to 10th percentile and 11th to 25th percentile were associated with a 0.76 percentage point higher (95%CI: 0.22-1.29, P=0.006) and 0.45 percentage point higher (95%CI: 0.02-0.88, P=0.04) hospital rate of respiratory failure compared to MIPS quality scores for orthopedic surgeons in the 51st to 100th percentile, respectively.

With the exception of thoracic surgeons, MIPS quality scores were not associated with significant changes in hospital rates of postoperative sepsis (Appendix Figure 4). MIPS quality scores for thoracic surgeons in the 1st to 10th percentile and 11th to 25th percentile were associated with a 0.45 (95%CI: 0.05-0.85, P=0.027) and a 0.38 (95%CI: 0.04-0.72, P=0.026) percentage point higher rate of postoperative sepsis compared to the reference population, respectively. Higher physician MIPS quality scores were not associated with lower hospital rates of postoperative acute kidney injury or postoperative bleeding (Appendix Figure 5 and 6).

© 2021 Glance LG et al. *JAMA Network Open.*

eFigure 1. Flowchart

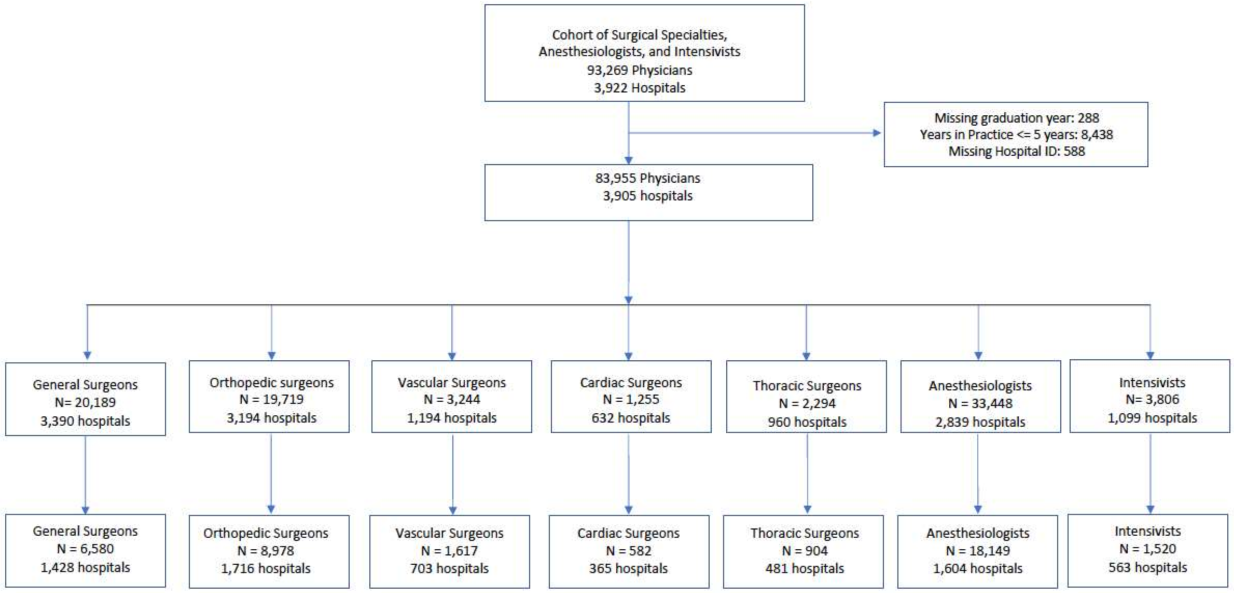

For each specialty group (e.g. general surgeons) within a hospital, we calculated the proportion of physicians who either (1) had quality scores equal to zero or were missing or (2) missing case volumes was equal to or greater than 20% were excluded from the analysis. Hospitals with 20% or more missing data (as defined above) were excluded from the analysis. For the purpose of this analysis, we assumed that physicians with MIPS quality scores of zero did not submit quality measures.

© 2021 Glance LG et al. *JAMA Network Open.*

eFigure 2. Distribution of Physician MIPS Quality Scores

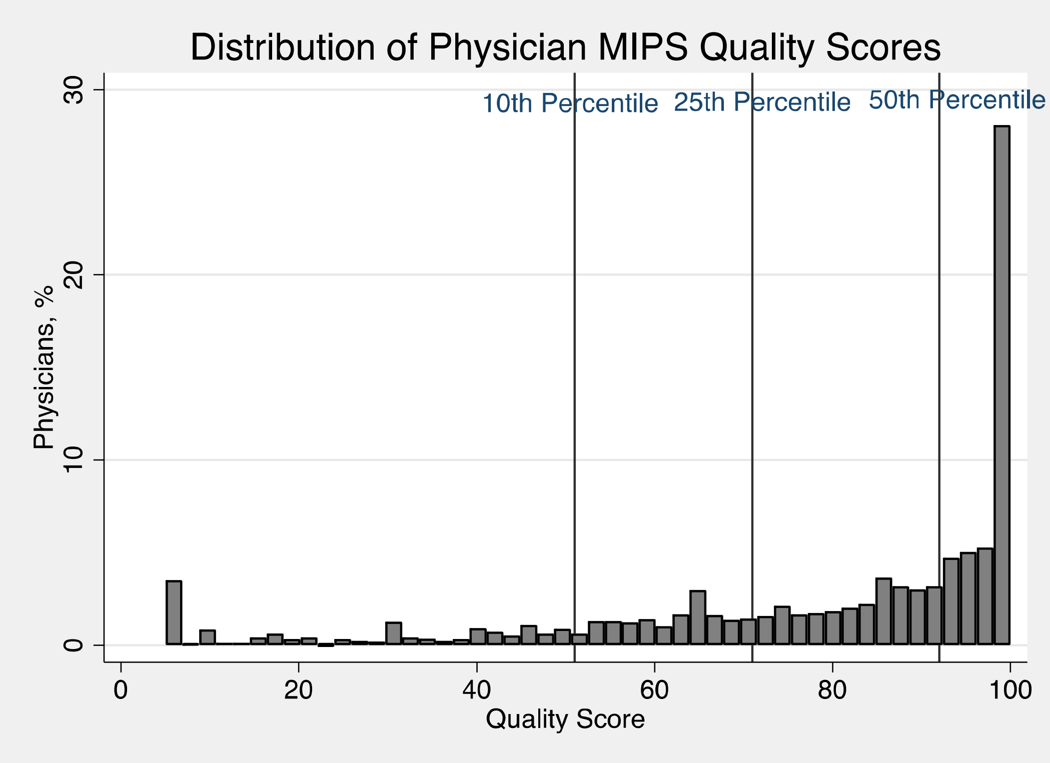

Physicians, as either individuals or groups of physicians, are evaluated in the MIPS using a composite score between 0 and 100 points based on quality, improvement activities, and promoting interoperability.

© 2021 Glance LG et al. *JAMA Network Open.*

eFigure 3. Association Between Physician Quality MIPS Scores and Postoperative Respiratory Failure

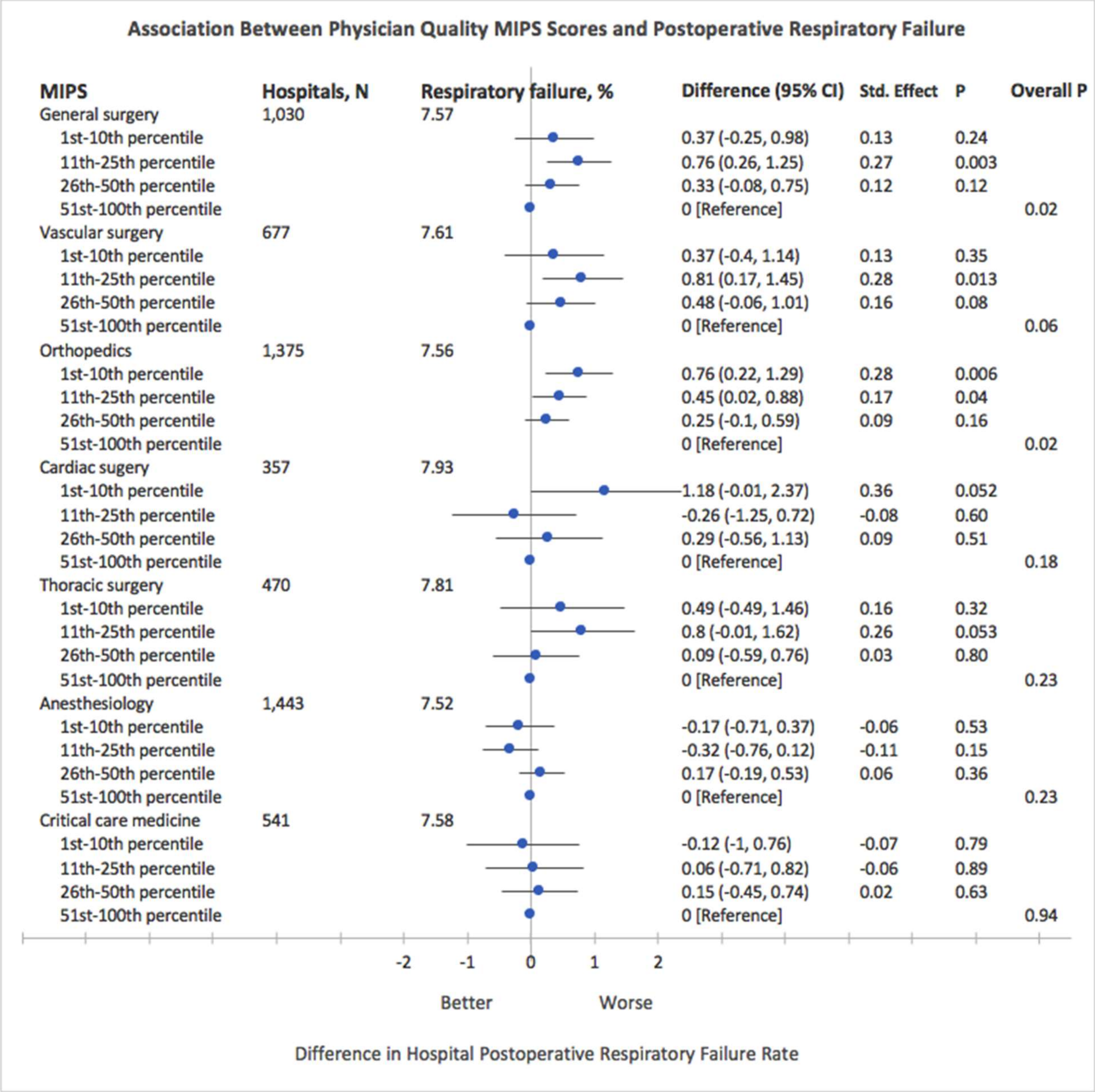

MIPS	Hospitals, N	Respiratory failure, %	Difference (95% CI)	Std. Effect	P	Overall P
General surgery	1,030	7.57				
1st-10th percentile			0.37 (-0.25, 0.98)	0.13	0.24	
11th-25th percentile			0.76 (0.26, 1.25)	0.27	0.003	
26th-50th percentile			0.33 (-0.08, 0.75)	0.12	0.12	
51st-100th percentile			0 [Reference]			0.02
Vascular surgery	677	7.61				
1st-10th percentile			0.37 (-0.4, 1.14)	0.13	0.35	
11th-25th percentile			0.81 (0.17, 1.45)	0.28	0.013	
26th-50th percentile			0.48 (-0.06, 1.01)	0.16	0.08	
51st-100th percentile			0 [Reference]			0.06
Orthopedics	1,375	7.56				
1st-10th percentile			0.76 (0.22, 1.29)	0.28	0.006	
11th-25th percentile			0.45 (0.02, 0.88)	0.17	0.04	
26th-50th percentile			0.25 (-0.1, 0.59)	0.09	0.16	
51st-100th percentile			0 [Reference]			0.02
Cardiac sugery	357	7.93				
1st-10th percentile			1.18 (-0.01, 2.37)	0.36	0.052	
11th-25th percentile			-0.26 (-1.25, 0.72)	-0.08	0.60	
26th-50th percentile			0.29 (-0.56, 1.13)	0.09	0.51	
51st-100th percentile			0 [Reference]			0.18
Thoracic surgery	470	7.81				
1st-10th percentile			0.49 (-0.49, 1.46)	0.16	0.32	
11th-25th percentile			0.8 (-0.01, 1.62)	0.26	0.053	
26th-50th percentile			0.09 (-0.59, 0.76)	0.03	0.80	
51st-100th percentile			0 [Reference]			0.23
Anesthesiology	1,443	7.52				
1st-10th percentile			-0.17 (-0.71, 0.37)	-0.06	0.53	
11th-25th percentile			-0.32 (-0.76, 0.12)	-0.11	0.15	
26th-50th percentile			0.17 (-0.19, 0.53)	0.06	0.36	
51st-100th percentile			0 [Reference]			0.23
Critical care medicine	541	7.58				
1st-10th percentile			-0.12 (-1, 0.76)	-0.07	0.79	
11th-25th percentile			0.06 (-0.71, 0.82)	-0.06	0.89	
26th-50th percentile			0.15 (-0.45, 0.74)	0.02	0.63	
51st-100th percentile			0 [Reference]			0.94

Difference refers to the percentage point difference between the MIPS group (e.g. 1st-10th percentile) and the reference category (51st to 100th percentile).

Abbreviations: CI – confidence interval; Std. effect – standardized effect refers to the standardized coefficient for each quartile such that a standardized coefficient of 1 for the 1st- 10th percentile indicates the percentage point difference between the 1st-10th percentile and the 51st-100th percentile is 1 standard deviation (based on the overall distribution of the hospital complication rate)

© 2021 Glance LG et al. *JAMA Network Open.*

eFigure 4. Association Between Physician Quality MIPS Scores and Postoperative Sepsis

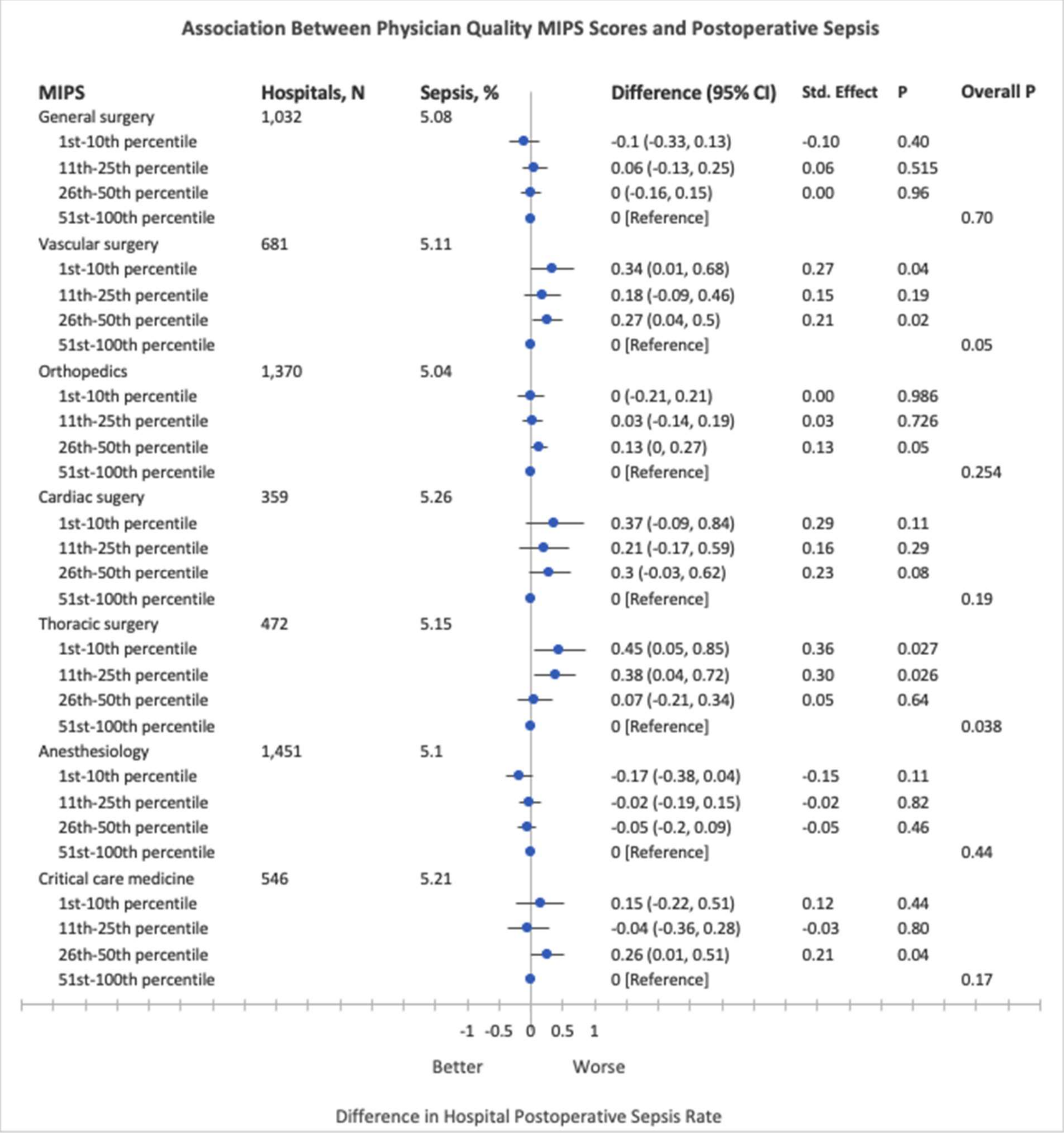

MIPS	Hospitals, N	Sepsis, %	Difference (95% CI)	Std. Effect	P	Overall P
General surgery	1,032	5.08				
1st-10th percentile			-0.1 (-0.33, 0.13)	-0.10	0.40	
11th-25th percentile			0.06 (-0.13, 0.25)	0.06	0.515	
26th-50th percentile			0 (-0.16, 0.15)	0.00	0.96	
51st-100th percentile			0 [Reference]			0.70
Vascular surgery	681	5.11				
1st-10th percentile			0.34 (0.01, 0.68)	0.27	0.04	
11th-25th percentile			0.18 (-0.09, 0.46)	0.15	0.19	
26th-50th percentile			0.27 (0.04, 0.5)	0.21	0.02	
51st-100th percentile			0 [Reference]			0.05
Orthopedics	1,370	5.04				
1st-10th percentile			0 (-0.21, 0.21)	0.00	0.986	
11th-25th percentile			0.03 (-0.14, 0.19)	0.03	0.726	
26th-50th percentile			0.13 (0, 0.27)	0.13	0.05	
51st-100th percentile			0 [Reference]			0.254
Cardiac sugery	359	5.26				
1st-10th percentile			0.37 (-0.09, 0.84)	0.29	0.11	
11th-25th percentile			0.21 (-0.17, 0.59)	0.16	0.29	
26th-50th percentile			0.3 (-0.03, 0.62)	0.23	0.08	
51st-100th percentile			0 [Reference]			0.19
Thoracic surgery	472	5.15				
1st-10th percentile			0.45 (0.05, 0.85)	0.36	0.027	
11th-25th percentile			0.38 (0.04, 0.72)	0.30	0.026	
26th-50th percentile			0.07 (-0.21, 0.34)	0.05	0.64	
51st-100th percentile			0 [Reference]			0.038
Anesthesiology	1,451	5.1				
1st-10th percentile			-0.17 (-0.38, 0.04)	-0.15	0.11	
11th-25th percentile			-0.02 (-0.19, 0.15)	-0.02	0.82	
26th-50th percentile			-0.05 (-0.2, 0.09)	-0.05	0.46	
51st-100th percentile			0 [Reference]			0.44
Critical care medicine	546	5.21				
1st-10th percentile			0.15 (-0.22, 0.51)	0.12	0.44	
11th-25th percentile			-0.04 (-0.36, 0.28)	-0.03	0.80	
26th-50th percentile			0.26 (0.01, 0.51)	0.21	0.04	
51st-100th percentile			0 [Reference]			0.17

Difference refers to the percentage point difference between the MIPS group (e.g. 1st-10th percentile) and the reference category (51st to 100th percentile).

Abbreviations: CI – confidence interval; Std. effect – standardized effect refers to the standardized coefficient for each quartile such that a standardized coefficient of 1 for the 1st- 10th percentile indicates the percentage point difference between the 1st-10th percentile and the 51st-100th percentile is 1 standard deviation (based on the overall distribution of the hospital complication rate)

© 2021 Glance LG et al. *JAMA Network Open.*

eFigure 5. Association Between Physician Quality MIPS Scores and Postoperative Acute Kidney Injury

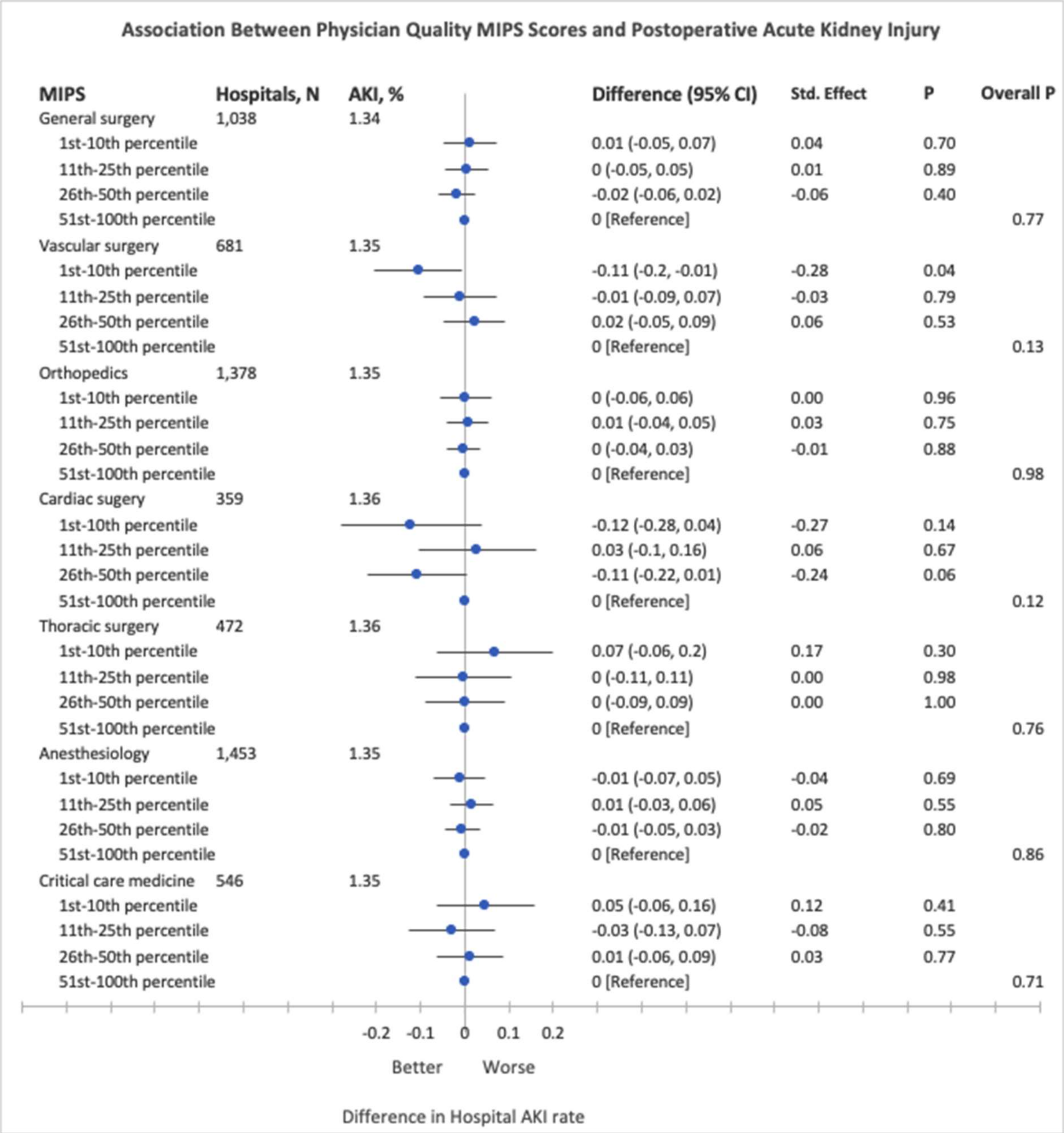

MIPS	Hospitals, N	AKI, %	Difference (95% CI)	Std. Effect	P	Overall P
General surgery	1,038	1.34				
1st-10th percentile			0.01 (-0.05, 0.07)	0.04	0.70	
11th-25th percentile			0 (-0.05, 0.05)	0.01	0.89	
26th-50th percentile			-0.02 (-0.06, 0.02)	-0.06	0.40	
51st-100th percentile			0 [Reference]			0.77
Vascular surgery	681	1.35				
1st-10th percentile			-0.11 (-0.2, -0.01)	-0.28	0.04	
11th-25th percentile			-0.01 (-0.09, 0.07)	-0.03	0.79	
26th-50th percentile			0.02 (-0.05, 0.09)	0.06	0.53	
51st-100th percentile			0 [Reference]			0.13
Orthopedics	1,378	1.35				
1st-10th percentile			0 (-0.06, 0.06)	0.00	0.96	
11th-25th percentile			0.01 (-0.04, 0.05)	0.03	0.75	
26th-50th percentile			0 (-0.04, 0.03)	-0.01	0.88	
51st-100th percentile			0 [Reference]			0.98
Cardiac sugery	359	1.36				
1st-10th percentile			-0.12 (-0.28, 0.04)	-0.27	0.14	
11th-25th percentile			0.03 (-0.1, 0.16)	0.06	0.67	
26th-50th percentile			-0.11 (-0.22, 0.01)	-0.24	0.06	
51st-100th percentile			0 [Reference]			0.12
Thoracic surgery	472	1.36				
1st-10th percentile			0.07 (-0.06, 0.2)	0.17	0.30	
11th-25th percentile			0 (-0.11, 0.11)	0.00	0.98	
26th-50th percentile			0 (-0.09, 0.09)	0.00	1.00	
51st-100th percentile			0 [Reference]			0.76
Anesthesiology	1,453	1.35				
1st-10th percentile			-0.01 (-0.07, 0.05)	-0.04	0.69	
11th-25th percentile			0.01 (-0.03, 0.06)	0.05	0.55	
26th-50th percentile			-0.01 (-0.05, 0.03)	-0.02	0.80	
51st-100th percentile			0 [Reference]			0.86
Critical care medicine	546	1.35				
1st-10th percentile			0.05 (-0.06, 0.16)	0.12	0.41	
11th-25th percentile			-0.03 (-0.13, 0.07)	-0.08	0.55	
26th-50th percentile			0.01 (-0.06, 0.09)	0.03	0.77	
51st-100th percentile			0 [Reference]			0.71

Difference refers to the percentage point difference between the MIPS group (e.g. 1st-10th percentile) and the reference category (51st to 100th percentile).

Abbreviations: CI – confidence interval; Std. effect – standardized effect refers to the standardized coefficient for each quartile such that a standardized coefficient of 1 for the 1st- 10th percentile indicates the percentage point difference between the 1st-10th percentile and the 51st-100th percentile is 1 standard deviation (based on the overall distribution of the hospital complication rate).

© 2021 Glance LG et al. *JAMA Network Open.*

eFigure 6. Association Between Physician Quality MIPS Scores and Postoperative Bleeding

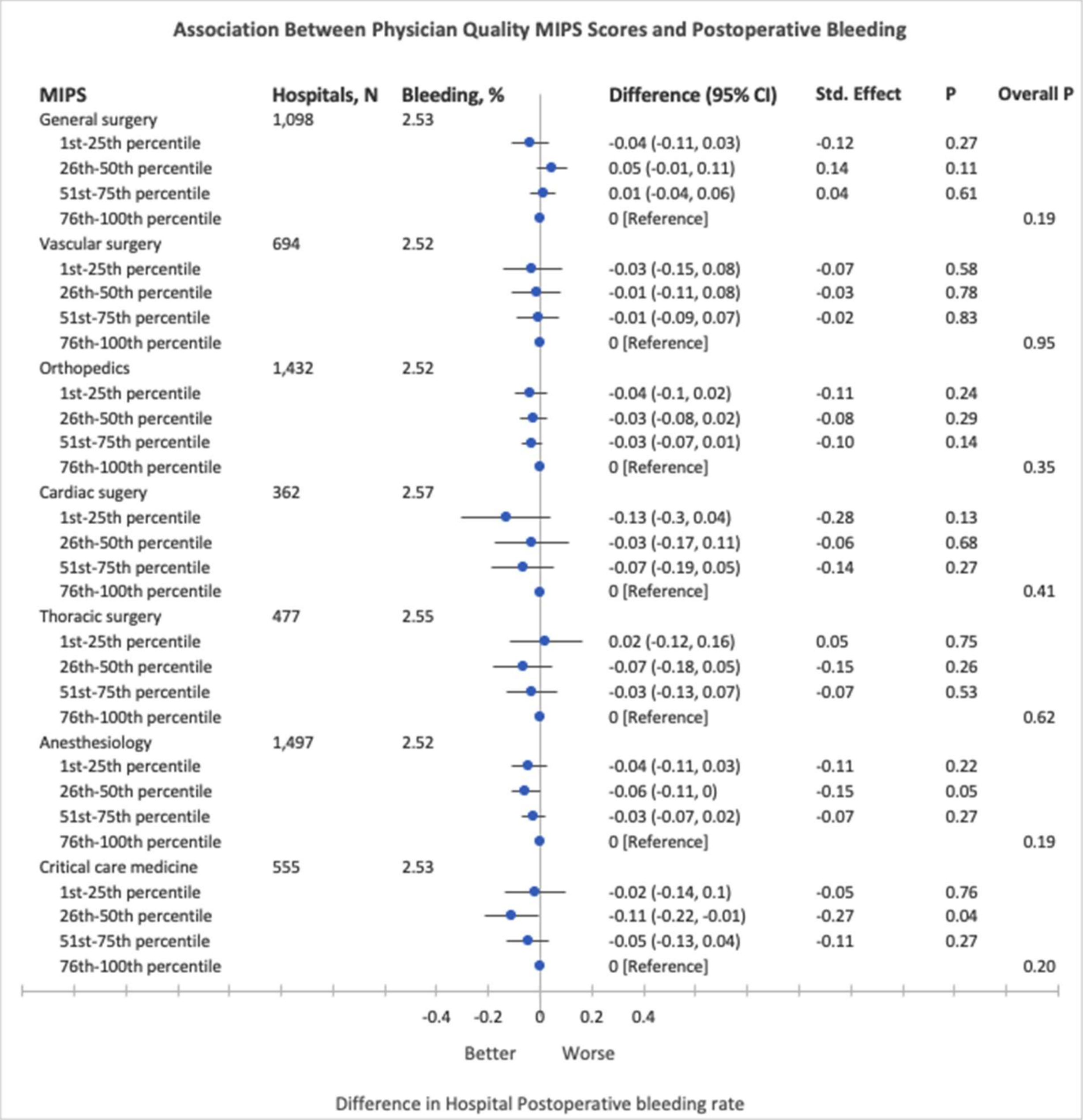

Difference refers to the percentage point difference between the MIPS group (e.g. 1st-10th percentile) and the reference category (51st to 100th percentile).

Abbreviations: CI – confidence interval; Std. effect – standardized effect refers to the standardized coefficient for each quartile such that a standardized coefficient of 1 for the 1st- 10th percentile indicates the percentage point difference between the 1st-10th percentile and the 51st-100th percentile is 1 standard deviation (based on the overall distribution of the hospital complication rate).

© 2021 Glance LG et al. *JAMA Network Open.*
